# Relationships of hematocrit concentration with dementia from a multiethnic population-based study

**DOI:** 10.3389/fnagi.2025.1543798

**Published:** 2025-02-14

**Authors:** David J. Roh, Minghua Liu, Kevin Strobino, Stephanie Assuras, Vanessa A. Guzman, Bonnie Levin, Steven L. Spitalnik, Tatjana Rundek, Clinton B. Wright, Mitchell S. V. Elkind, Jose Gutierrez

**Affiliations:** ^1^Department of Neurology, Vagelos College of Physicians and Surgeons, Columbia University, New York, NY, United States; ^2^Department of Neurology, University of Miami, Miami, FL, United States; ^3^Department of Pathology and Cell Biology, Vagelos College of Physicians and Surgeons, Columbia University, New York, NY, United States; ^4^National Institute of Neurological Disorders and Stroke, Bethesda, MD, United States; ^5^Department of Epidemiology, Mailman School of Public Health, Columbia University, New York, NY, United States

**Keywords:** red blood cell, hematocrit, cognition, dementia, epidemiology, cerebral small vessel disease

## Abstract

**Objective:**

Red blood cell (RBC) concentration impacts cerebrovascular disease, yet it is unclear whether RBC concentrations relate to dementia risk, particularly in racially/ethnically diverse cohorts. We investigated whether RBC concentrations associate with incident dementia risk in a diverse population of stroke-free individuals and explored whether cerebral small vessel disease (CSVD) mediates this relationship.

**Methods:**

A longitudinal observational analysis was performed using a population-based cohort of stroke-free, older adult participants (>50 years) from the Northern Manhattan Study (NOMAS) enrolled between 2003 and 2008. Participants received baseline hematocrit testing, MRI neuroimaging, and cognitive assessments at baseline and long-term follow-up. Associations of baseline hematocrit as a categorical variable (low, normal [reference], and high based on laboratory reference levels) with incident dementia were assessed using Cox models adjusting for relevant covariates. Separate analyses investigated whether MRI CSVD mediated these relationships.

**Results:**

We studied 1,207 NOMAS participants (mean age 71 ± 9 years, 60% female, 66% Hispanic). Mean hematocrit was 41.2% (±3.8) with 16% of participants developing incident dementia. Lower hematocrit associated with increased dementia risk (adjusted hazard ratio 1.81 [1.01–3.23]) after adjusting for age, sex, race/ethnicity, education, APOE status, and comorbidities. High hematocrit was not associated with dementia risk. No interactions by sex or race/ethnicity were seen and baseline CSVD did not mediate relationships between hematocrit and dementia.

**Conclusion:**

Low hematocrit associated with dementia risk in our diverse population cohort. However, our study limitations in laboratory and neuroimaging timing in addition to clarifying mechanistic underpinnings for our observations necessitates further work to clarify whether anemia can serve as a trackable, preventable/treatable risk factor for dementia.

## Introduction

Red blood cell (RBC) concentrations are known to impact cerebrovascular disease incidence and outcomes. This relationship appears to also apply to dementia as low RBC concentrations/anemia, a prevalent condition in the elderly (Guralnik et al., 2004), has been identified as an independent risk factor for incident dementia (Hong et al., 2013). However, separate studies have identified that these relationships exist across a range of RBC concentrations, specifically with both low and high RBC concentration extremes associating with dementia risk (Wolters et al., 2019; Shah et al., 2011). It is unclear whether these findings are generalizable to multi-ethnic communities who have different risks for both anemia and polycythemia. Furthermore, underlying mechanisms for these relationships are unknown. It is currently posited that both low and high RBC concentrations could directly play a role through hypoxic/ischemic and microthrombotic cerebral insults, respectively (Walton et al., 2017; Kurtz et al., 2010; Oddo et al., 2012). We and others have separately identified that both low and high RBC concentrations associate with asymptomatic, covert cerebral small vessel disease across a variety of disease conditions (Roh et al., 2024; Roh et al., 2023; Grotemeyer et al., 2014; Kishimoto et al., 2020; Kiyohara et al., 1986; Gotoh et al., 2015; van der Veen et al., 2015; Wood and Kee, 1985), creating a premise that ischemia or microthrombosis may indeed play a role in dementia risk. While it is known that neuroimaging evidence of covert cerebrovascular disease increases the risk of dementia (Vermeer et al., 2003; Smith et al., 2017; Rizvi et al., 2021; Wright et al., 2008), it remains to be determined whether this mediates associations of RBC concentration and dementia. Thus, we sought to investigate the hypothesis that RBC concentration is related to incident dementia risk and that these relationships would be mediated by covert cerebral small vessel disease in a multi-ethnic, stroke-free, population-based cohort study.

## Methods

### Northern Manhattan study

We included NOMAS participants enrolled in an MRI sub-study between 2003 and 2008.

**Figure 1:**
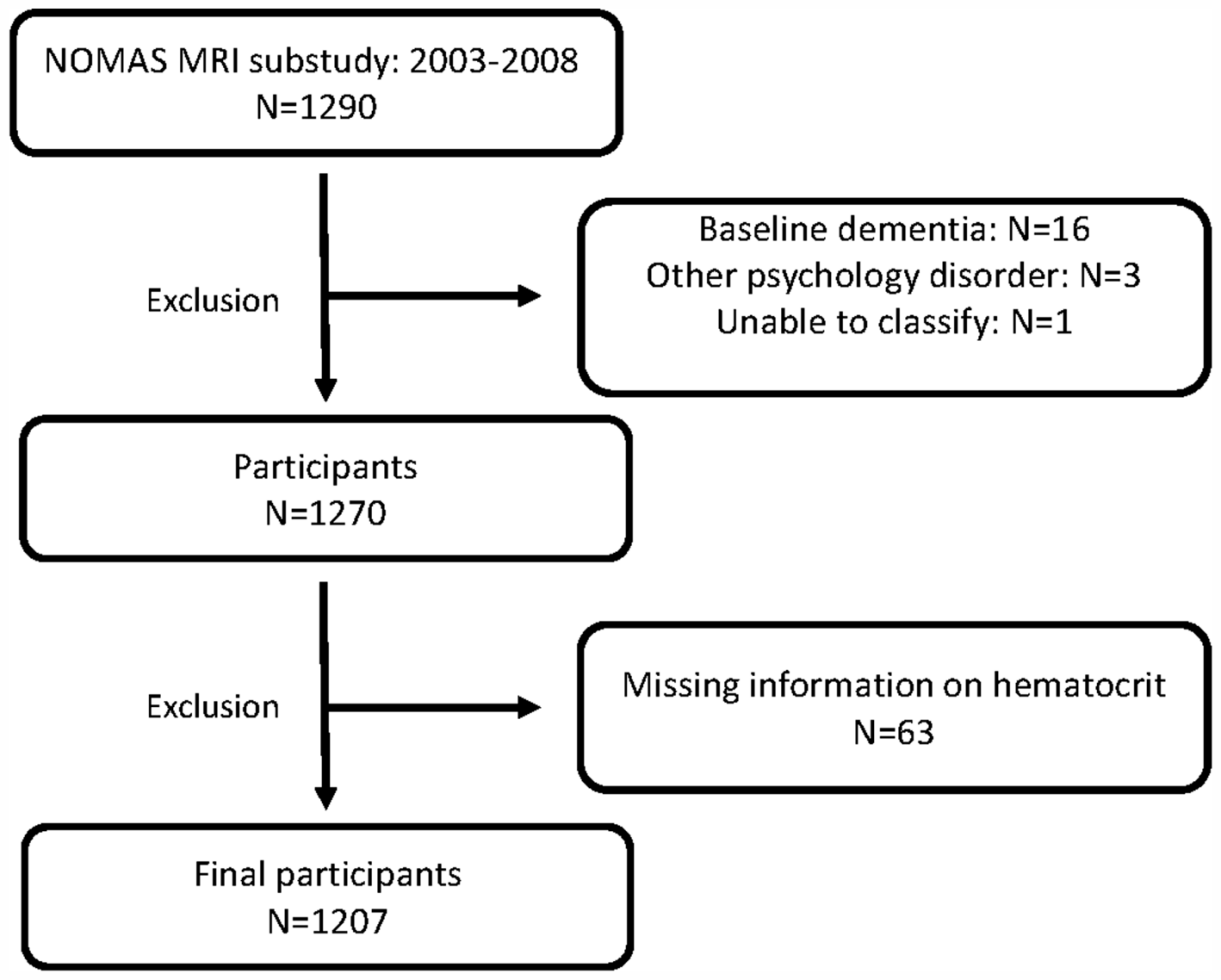
Patient inclusion and exclusion. NOMAS, Northern Manhattan Study, MRI, magnetic resonance imaging.

Participants enrolled were stroke-free individuals over 50 years old from the Northern Manhattan community that were either NOMAS participants or unrelated household members (Gutierrez et al., 2015; Wright et al., 2021). Participants with available baseline RBC laboratory assessments, MRI imaging, and follow-up cognitive assessments were assessed. Participants with dementia at baseline were excluded from analyses (Figure 1).

#### RBC concentration assessment

Hematocrit at the baseline visit was used as the assessment of RBC concentration. Given potential non-linear relationships of hematocrit with outcomes of interest, hematocrit was primarily assessed as a categorical variable based on reference ranges of the NOMAS core laboratory (normal vs. low vs. high [male normal range 37–48%; female normal range 34–43%]). Hematocrit was secondarily assessed as a continuous exposure variable. Separate analyses were performed using hemoglobin as a surrogate assessment of RBC concentration. Hemoglobin was assessed as a categorical variable similarly based on laboratory reference ranges (male normal range: 12.6–17 g/dL; female normal range: 12–15.8 g/dL).

#### Cognitive assessments and outcome definition

Participants underwent structured questionnaires and neuropsychological assessments by trained research staff in either English or Spanish to assess cognitive function as previously described (Wright et al., 2021). Assessments occurred at baseline with two additional follow-up visits at roughly 5-year intervals. Incident dementia was assessed as our primary outcome. Dementia was adjudicated by a multidisciplinary team, including a neurologist and a neuropsychologist, by consensus. Criteria for dementia was based off of criteria from the National Institute on Aging-Alzheimer’s Association based on (1) evidence of decline from prior level of performance in at least 1 cognitive domain, (2) functional impairment, and (3) lack of psychiatric or other diagnosis that would explain the cognitive status as previously described (Wright et al., 2021). Diagnosis was based on available clinical data including repeated neuropsychological testing, functional assessments (Informant Questionnaire of Cognitive Decline in the Elderly, Older Americans Resources and Services IADLs, Functional Assessment Scale and/or Clinical Dementia Rating [CDR] score), annual Telephone Interview for Cognitive Status (TICS) score, annual medication history (including newly self-reported dementia medications), self-reported diagnosis of dementia or self-reported “cognitive failures,” and medical records from participants who followed at our institution for their care (Wright et al., 2021).

#### MRI neuroimaging

Baseline standardized MRIs were obtained for these NOMAS participants on a dedicated 1.5 T research MRI (Philips Medical Systems). MRI acquisition was performed concurrently with baseline hematocrit laboratory assessments. A composite assessment of small vessel disease severity was utilized (severe: presence of 2–4 small vessel disease markers vs. non-severe: 0–1 small vessel disease markers). Secondarily, we separately assessed presence of chronic, covert lacunar ischemic infarcts (asymptomatic strokes), severe small perivascular space burden (SPVS, defined as upper quintile), presence of severe white matter hyperintensity burden (defined as upper quintile), and cerebral microbleeds. These markers were assessed using previously described methods via a central neuroimaging analysis core blinded to study outcomes (Gutierrez et al., 2015).

#### Statistical analysis

Participant characteristics were compared among low, normal, and high range hematocrit. Relationships of hematocrit, defined as a categorical variable (low, normal, high) with incident dementia were assessed in primary analyses using Cox regression models. Supremum tests were performed to ensure proportional hazard assumptions were met. Additional models were assessed analyzing hematocrit as a continuous variable. Given potential non-linear associations of hematocrit with dementia (Wolters et al., 2019; Shah et al., 2011), separate models for hematocrit as a continuous variable were performed using restricted cubic splines regression fitting. Fine and Gray regression was performed to calculate the cumulative incidence risk of dementia per hematocrit categorical group while addressing the competing risk of death. In secondary analyses, we assessed whether covert small vessel disease on MRI mediated the relationship of hematocrit with incident dementia. All models were adjusted for age, sex, race/ethnicity, hypertension, dyslipidemia, diabetes, smoking status, renal function (as assessed using eGFR), education attainment, APOE status, and total cranial volume. We investigated statistical interactions by sex, race/ethnicity, and APOE status (APOEe4 allele carrier status was assessed by Hha1 digestion of PCR products amplified form genomic DNA). Finally, mediation analyses were performed to estimate whether small vessel disease neuroimaging markers were the mediator for hematocrit’s relationship with dementia. Specifically, hematocrit was defined as the exposure and dementia was the outcome with the following assessed as mediators: composite small vessel disease, lacunar infarcts, white matter hyperintensities, and SPVS. A logistic regression model was used to explore the association of exposure and outcome, mediators and outcome, exposure and composite small vessel disease, lacunar infarcts, white matter hyperintensities, and SPVS separately. Statistical significance was assessed at *p*-value <0.05. Analyses were performed using SAS (version 9.4, SAS Institute, Cary, NC).

#### Standard protocol approvals and patient consents

The NOMAS protocol was approved by the Institutional Review Boards at Columbia University Irving Medical Center and the University of Miami. Informed consent was obtained from all participants.

## Results

Data from 1,207 stroke-free participants from NOMAS with available hematocrit and cognitive outcomes were assessed (Figure 1). Baseline characteristics are shown in Table 1. The mean age of our cohort was 71, 60% were female, and 66% were Hispanic. The mean hematocrit was 41.2%. The majority of participants had hematocrits within the normal reference range (83%) with low and high hematocrit identified in 5 and 12%, respectively. We identified that participants with low hematocrit were more likely to be older, non-Hispanic Black, and diabetic compared to normal and high hematocrit participants (Table 1). There were no notable intergroup differences in baseline cognitive assessments, or years of education between low, normal, and high hematocrit groups.

**Table 1:** Baseline characteristics of NOMAS participants with normal, low, and high hematocrit.

	Overall NOMAS cohort *N* = 1,207	Normal Hct *N* = 1,000	Low Hct *N* = 59	High Hct *N* = 148	*p*-value
Age: mean (SD)	70.5 (8.9)	70.3 (9.0)	73.1 (8.5)	70.4 (8.6)	0.055
Female: *N* (%)	722 (59.8)	570 (57.0)	32 (54.2)	120 (81.1)	<0.001
Race: *N* (%)					0.002
Non-Hispanic White	205 (17.0)	172 (17.2)	10 (16.9)	23 (15.5)	
Non-Hispanic Black	203 (16.8)	162 (16.2)	21 (35.6)	20 (13.5)	
Hispanic	799 (66.2)	666 (66.6)	28 (47.5)	105 (70.9)	
Medical history: *N* (%)					
Hypertension	947 (78.5)	776 (81.9)	50 (84.7)	121 (81.8)	0.250
Diabetes	309 (25.6)	260 (26.0)	24 (40.7)	25 (16.9)	0.001
Dyslipidemia	977 (80.9)	806 (80.6)	52 (88.1)	119 (80.4)	0.353
Smoking: *N* (%)	141 (11.7)	107 (10.7)	7 (11.97)	27 (18.2)	0.029
Hematocrit (%): mean (SD)	41.2 (3.8)	41.1 (3.0)	33.2 (2.2)	45.8 (2.8)	<0.001
Hemoglobin (g/dL): mean (SD)	13.6 (1.5)	13.6 (1.2)	10.8 (1.0)	15.1 (1.5)	<0.001
Cognitive assessments:					
Number of assessments: median (IQR)	2 (2–3)	2 (2–3)	2 (1–2)	2 (2–3)	0.002
Time to first follow-up (years): median (IQR)	0.93 (0.78–1.09)	0.93 (0.77–1.09)	0.94 (0.8–1.06)	0.91 (0.80–1.06)	0.515
Follow up (years): median (IQR)	9.6 (5.1–12.5)	10.4 (5.1–12.6)	5.1 (4.9–10.7)	5.7 (5.1–12.3)	0.001
Years of education: mean (SD)	9.7 (5.2)	9.7 (5.2)	10.5 (5.1)	9.1 (5.0)	0.157
Baseline cognitive domain Z-score: mean (SD)	−0.006 (0.718)	−0.001 (0.725)	0.019 (0.643)	−0.049 (0.70)	0.727
Baseline MMSE: mean (SD)	26.7 (3.3)	26.8 (3.2)	27.3 (3.2)	26.4 (3.6)	0.197
Incident Dementia: *N* (%)	188 (15.6)	152 (15.2)	14 (23.7)	22 (14.9)	0.207
Baseline MRI findings: *N* (%)					
Chronic covert lacunar infarct	214 (17.7)	175 (17.5)	13 (22.0)	26 (17.6)	0.674
Small perivascular space	74 (6.1)	64 (6.4)	4 (6.8)	6 (4.1)	0.528

NOMAS, Northern Manhattan Study; SD, standard deviation; Hct, hematocrit; MMSE, mini mental state evaluation.

**Figure 2:**
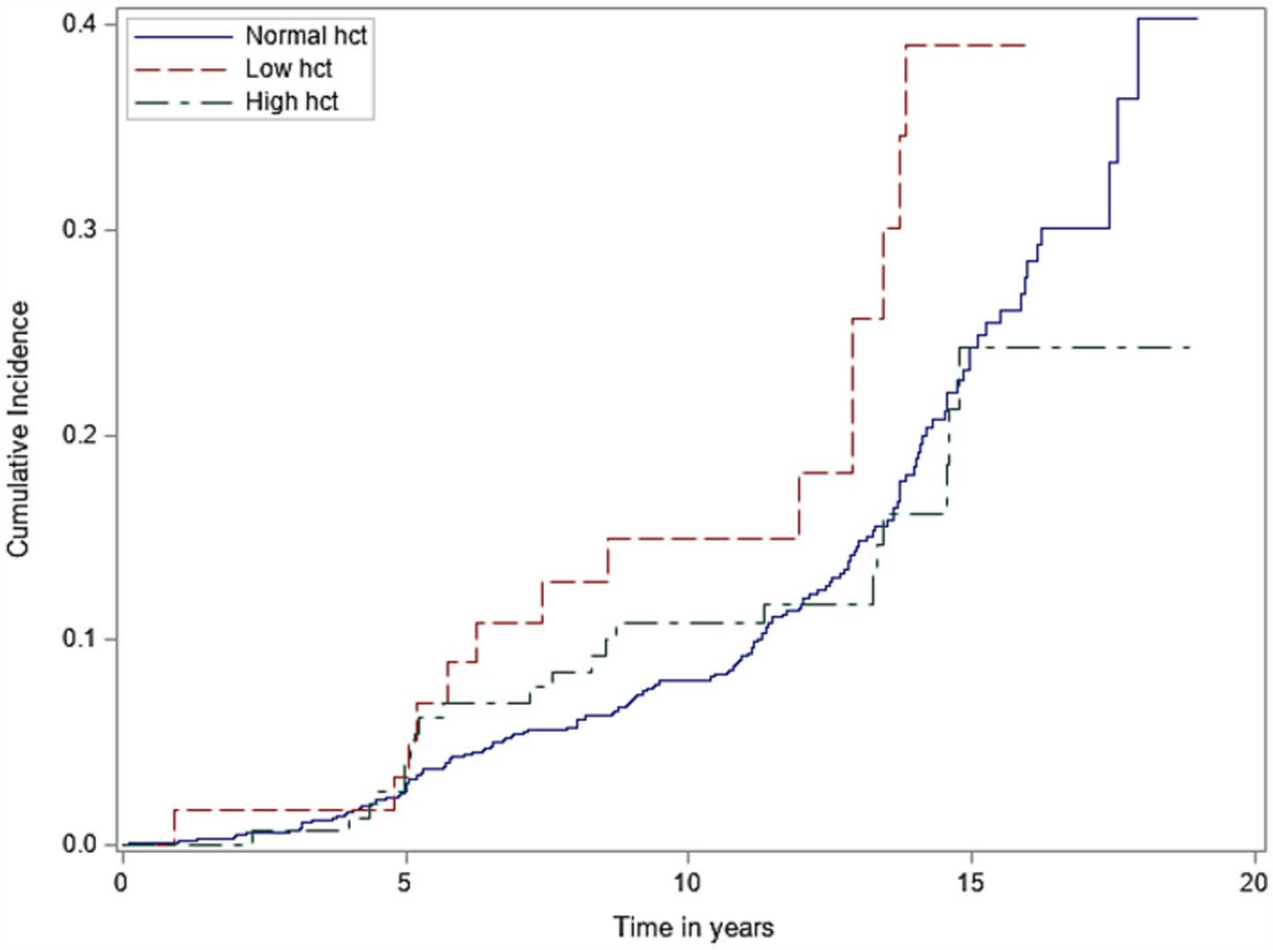
Cumulative incidence of dementia by different hematocrit groups (low vs. normal vs. high). Hct, hematocrit.

Among our entire cohort, incident dementia was adjudicated in 16% over a median total follow-up time of 9.6 years. When assessing hematocrit as a categorical variable (normal, low, and high) using laboratory reference ranges set by our centralized laboratory, we identified that patients with low hematocrit had an increased risk of incident dementia when referenced to participants with normal hematocrit concentrations (adjusted hazard ratio 1.81 [1.01–3.23], *p* = 0.04). No associations of high hematocrit category with dementia (adjusted hazard ratio 0.70 [0.43–1.15], *p* = 0.16) were seen (Supplementary Table 1a). When assessing the relationship of baseline hematocrit as a continuous linear variable with incident dementia, we again identified that lower hematocrit associated with increased risk of dementia (adjusted hazard ratio 0.95 per 1% change in hematocrit [0.91–0.99]; *p* = 0.04). We did not identify significant U-shaped relationships of hematocrit extremes with incident dementia (chi-square: 2.08; *p* = 0.56), but it was notable that these relationships were non-linear (Supplementary Figure 1). The Fine and Gray test identified that participants with lower hematocrits had higher probability of dementia over shorter periods of follow-up time compared to normal and high hematocrit groups, but these differences were not statistically significant (*p* = 0.20; see Figure 2). No interactions between hematocrit and sex, race/ethnicity, or APOE4 status were seen (Supplementary Table 2). Separate models utilizing hemoglobin instead of hematocrit as the assessment of RBC concentration yielded similar associations of low, but not high, hemoglobin categories with incident dementia risk (low hemoglobin adjusted HR 1.54 [1.04–2.28], *p* = 0.03; high hemoglobin adjusted HR 0.70 [0.17–2.87], *p* = 0.62; Supplementary Table 1b).

In our MRI analyses of baseline chronic, covert cerebral small vessel disease markers, we identified severe cerebral small vessel disease burden in 12% of the overall cohort. Assessments of individual cerebral small vessel disease markers by hematocrit categories can be seen in Table 2. We identified that participants with low hematocrit were more likely to have baseline MRI evidence of white matter disease burden, however differences were non-significant via chi-square testing. In our mediation analyses, we did not identify clear evidence that either composite severe small vessel disease presence or separate, individual small vessel disease markers (chronic covert lacunar ischemic infarcts, SPVS, white matter hyperintensity, cerebral microbleeds) mediated the relationship of low hematocrit (or low hemoglobin; data not shown) with incident dementia (Supplementary Figure 2).

**Table 2:** Prevalence of chronic covert cerebral small vessel disease MRI markers by hematocrit group.

	Overall NOMAS cohort	Normal Hct	Low Hct	High Hct	*p*-value
Lacunar infarct	74 (6)	64 (6)	4 (7)	6 (4)	0.53
Small perivascular space	220 (19)	180 (19)	9 (16)	31 (22)	0.59
White matter hyperintensity	232 (19)	187 (19)	17 (29)	28 (19)	0.16
Cerebral microbleed	89 (8)	69 (7)	6 (10)	14 (10)	0.46
Severe composite small vessel disease	129 (12)	103 (11)	9 (16)	17 (12)	0.55

NOMAS, Northern Manhattan Study; Hct, hematocrit.

## Discussion

In our large population-based study of ethnically diverse, non-stroke participants over 50 years old with long-term follow-up, we identified independent relationships of lower hematocrit with incident dementia after adjusting for known risk factors of dementia. While we evaluated hematocrit across its entire range, it was notable that we did not identify additional relationships of higher hematocrit with dementia. And despite our prior published findings identifying relevant relationships of lower hematocrit concentrations with MRI evidence of asymptomatic, covert cerebral small vessel disease (Roh et al., 2024), these MRI biomarkers did not appear to be primary drivers/mediators for low hematocrit’s relationship with dementia.

Previous population cohorts have reported U-shaped relationships of RBC concentrations at both extremes (i.e., anemia and relative polycythemia) with incident dementia (Wolters et al., 2019; Shah et al., 2011). Yet, despite having similar dementia prevalence in our cohort compared to these prior studies, we were only able to replicate lower hematocrit’s relationship with dementia. It is worth noting that prior studies which identified U-shaped relationships of both RBC concentration extremes with dementia did see greater effect estimates for dementia risk in lower RBC concentration groups compared to higher ones (Wolters et al., 2019; Shah et al., 2011). Thus, our data may add to this literature by providing evidence that lower hematocrit plays a more pervasive, generalizable role than higher hematocrit on dementia risk. While we did not identify relevant interactions of hematocrit with race/ethnicity and adjusted for baseline medical disease characteristics, it could be posited that our diverse cohort, comprised primarily of Hispanic participants with higher prevalence of baseline cardiac and cerebrovascular risk factors is more vulnerable to the impacts of low RBC concentrations. Prior multi-ethnic cohorts of Black participants have reported similar anemia-dementia relationships without interactions of race and anemia (Hong et al., 2013).

Current mechanisms and drivers for lower RBC concentration’s relationship to dementia remains unclear (Guralnik et al., 2004; Hong et al., 2013; Wolters et al., 2019). Because RBCs are critical for cerebral oxygen delivery, it has been posited that anemia creates risk for cerebral hypoxia, inflammation, and even ischemia, all factors that may lead to neuronal injury and dementia. To this extent, we had previously identified in a cross-sectional analysis of our NOMAS cohort that lower hematocrit concentrations associate with increased risk of chronic MRI cerebrovascular disease markers: covert lacunar infarcts and SPVS. Though this supported the idea that low hematocrit’s relationship with dementia could be mediated by asymptomatic, covert neuroimaging biomarkers of cerebral small vessel disease, this was not recapitulated in our mediation analyses. This could suggest that anemia’s impact on dementia is unrelated to covert cerebral small vessel disease burden as other studies (albeit in participants of primarily European descent) did not identify that anemia relates to covert infarcts. Instead, these studies identified that anemia has overlapping relevance with cerebral perfusion (unavailable in our study), an MRI neuroimaging biomarker known to be a risk factor for dementia (Wolters et al., 2019; Wolters et al., 2017). But it should be noted that our baseline MRI acquisition was performed in conjunction with our hematocrit assessments, which preceded incident dementia diagnosis by several years. Thus, it is possible that there still may be a mediating role of covert cerebral small vessel disease on our findings that simply was not identified given our study’s inherent concurrent baseline laboratory/MRI assessment design. It is feasible that dynamic changes in covert cerebral small vessel disease burden occurs over time in anemic patients which would be better assessed with serial longitudinal MRI imaging, which was unavailable in our dataset. Because mediation analyses necessitate a temporal ordering of exposure variable, mediator, or outcome, our study’s acquisition of exposure (hematocrit) and mediator (MRI neuroimaging) variables at concurrent times may have limited the accuracy of these analyses. Furthermore, mediation analyses necessitate rigorous accounting for confounders, thus observational designs may have inherent limitations in disentangling anemia’s direct causal impact on cerebral small vessel disease and dementia. Finally, given our exclusion of patients with symptomatic strokes, we may have failed to identify relevant mediating effects given the lack of generalizability of our data to patients who had encountered symptomatic ischemic stroke. Thus, future observational and translational work will be needed to assess whether anemia is an upstream risk factor that can lead to differential changes in cerebral small vessel disease burden over time, thereby impacting dementia risk.

Our study strengths included the analysis of a large, prospective population-based cohort of ethnically diverse participants, the long-term follow-up of cognitive assessments across the study course, the use of standardized MRI neuroimaging analyses, and adjustment for relevant covariates for dementia. However, several study limitations require mention. First and foremost, our study may have been subject to unmeasured confounding. While we adjusted for traditional risk factors in all models including confounders for hematocrit’s relationship with dementia [including renal function, which has previously been identified as a relevant risk factor for dementia (Weiner et al., 2017; Khatri et al., 2009)], we did not have data on etiologic drivers for low hematocrit/anemia (i.e., inflammation, iron deficiency, nutritional status, impaired erythropoiesis). Additionally, we did not have data on additional blood count parameters (assessments of RBC size/volume) or assessments of immature erythrocytes/RBC indices (Shah et al., 2011; Walton et al., 2017). Because certain drivers for anemia are also known to relate to dementia risk, it remains unclear whether low hematocrit’s relationship with dementia was driven by hematocrit itself or underlying drivers for anemia development. Further studies assessing hematocrit as well as underlying drivers for anemia will allow for a deeper understanding of mechanistically causal relationships that can be either used as screening risk factors or even targets for dementia prevention. Second, it similarly remains uncertain whether and how low hematocrit (or its underlying drivers) directly cause dementia. Our MRI data did not appear to suggest that cerebral small vessel disease mediates this relationship. However, as previously stated, further work assessing serial neuroimaging as well as other MRI neuroimaging markers (i.e., cerebral perfusion and connectivity) will be helpful in clarifying how low hematocrit impacts brain health. Similarly, it is possible that more advanced/higher resolution MRI neuroimaging, not available in our study, may provide additional insights into small vessel disease markers’ impact on our observed relationships. Additionally, human observational data will need to be paired with translational and pre-clinical models to disentangle the natural overlap of low hematocrit concentrations and underlying medical disease to establish causal relationships of low hematocrit and dementia. Lastly, our study was limited to hematocrit as the assessment of RBC concentration and RBC characteristics. It is unclear whether other factors related to the RBC number, size, maturity, or deformability of the RBC itself could play a role in dementia risk.

## Conclusion

We identified relationships between low hematocrit concentrations with incident dementia risk in an ethnically diverse population-based study. The biological underpinnings behind these findings are unknown. Further investigation into RBC concentrations’ impact on neuroimaging markers of brain health and dementia risk is required to assess whether this can be leveraged as a screening tool or even modifiable treatment target.

## Data Availability

The raw data supporting the conclusions of this article will be made available by the authors, without undue reservation.

## References

[ref1] Gotoh S. Hata J. Ninomiya T. Hirakawa Y. Nagata M. Mukai N.. (2015). Hematocrit and the risk of cardiovascular disease in a Japanese community: the Hisayama study. Atherosclerosis 242, 199–204. doi: 10.1016/j.atherosclerosis.2015.07.014 26204496

[ref2] Grotemeyer K. C. Kaiser R. Grotemeyer K.-H. Husstedt I. W. (2014). Association of elevated plasma viscosity with small vessel occlusion in ischemic cerebral disease. Thromb. Res. 133, 96–100. doi: 10.1016/j.thromres.2013.10.028 24238841

[ref3] Guralnik J. M. Eisenstaedt R. S. Ferrucci L. Klein H. G. Woodman R. C. (2004). Prevalence of anemia in persons 65 years and older in the United States: evidence for a high rate of unexplained anemia. Blood 104, 2263–2268. doi: 10.1182/blood-2004-05-1812 15238427

[ref4] Gutierrez J. Elkind M. S. V. Cheung K. Rundek T. Sacco R. L. Wright C. B. (2015). Pulsatile and steady components of blood pressure and subclinical cerebrovascular disease: the northern Manhattan study. J. Hypertens. 33, 2115–2122. doi: 10.1097/HJH.0000000000000686 26259124 PMC4871260

[ref5] Hong C. H. Falvey C. Harris T. B. Simonsick E. M. Satterfield S. Ferrucci L.. (2013). Anemia and risk of dementia in older adults: findings from the health ABC study. Neurology 81, 528–533. doi: 10.1212/WNL.0b013e31829e701d 23902706 PMC3775683

[ref6] Khatri M. Nickolas T. Moon Y. P. Paik M. C. Rundek T. Elkind M. S. V.. (2009). CKD associates with cognitive decline. J. Am. Soc. Nephrol. 20, 2427–2432. doi: 10.1681/ASN.2008101090 19729443 PMC2799177

[ref7] Kishimoto S. Maruhashi T. Kajikawa M. Matsui S. Hashimoto H. Takaeko Y.. (2020). Hematocrit, hemoglobin and red blood cells are associated with vascular function and vascular structure in men. Sci. Rep. 10:11467. doi: 10.1038/s41598-020-68319-1 32651430 PMC7351756

[ref8] Kiyohara Y. Ueda K. Hasuo Y. Fujii I. Yanai T. Wada J.. (1986). Hematocrit as a risk factor of cerebral infarction: long-term prospective population survey in a Japanese rural community. Stroke 17, 687–692. doi: 10.1161/01.STR.17.4.687, PMID: 3738953

[ref9] Kurtz P. Schmidt J. M. Claassen J. Carrera E. Fernandez L. Helbok R.. (2010). Anemia is associated with metabolic distress and brain tissue hypoxia after subarachnoid hemorrhage. Neurocrit. Care. 13, 10–16. doi: 10.1007/s12028-010-9357-y 20383611

[ref10] Oddo M. Levine J. M. Kumar M. Iglesias K. Frangos S. Maloney-Wilensky E.. (2012). Anemia and brain oxygen after severe traumatic brain injury. Intensive Care Med. 38, 1497–1504. doi: 10.1007/s00134-012-2593-1 22584800

[ref11] Rizvi B. Lao P. J. Chesebro A. G. Dworkin J. Amarante E. Beato J.. (2021). Association of Regional White Matter Hyperintensities with Longitudinal Alzheimer-like Pattern of neurodegeneration in older adults. JAMA Netw. Open 4:e2125166. doi: 10.1001/jamanetworkopen.2021.25166, PMID: 34609497 PMC8493439

[ref12] Roh D. J. Boehme A. Mamoon R. Hooper D. Cottarelli A. Ji R.. (2023). Relationships of hemoglobin concentration, ischemic lesions, and clinical outcomes in patients with intracerebral hemorrhage. Stroke 54, 1021–1029. doi: 10.1161/STROKEAHA.122.041410 36779340 PMC10050127

[ref13] Roh D. J. Murguia-Fuentes R. Gurel K. Khasiyev F. Rahman S. Bueno P. P.. (2024). Relationships of hematocrit with chronic covert and acute symptomatic lacunar ischemic lesions. Neurology 102:e207961. doi: 10.1212/WNL.0000000000207961 38165319 PMC10870744

[ref14] Shah R. C. Buchman A. S. Wilson R. S. Leurgans S. E. Bennett D. A. (2011). Hemoglobin level in older persons and incident Alzheimer disease: prospective cohort analysis. Neurology 77, 219–226. doi: 10.1212/WNL.0b013e318225aaa9 21753176 PMC3136057

[ref15] Smith E. E. Saposnik G. Biessels G. J. Doubal F. N. Fornage M. Gorelick P. B.. (2017). Prevention of stroke in patients with silent cerebrovascular disease: a scientific statement for healthcare professionals from the American Heart Association/American Stroke Association. Stroke 48, e44–e71. doi: 10.1161/STR.0000000000000116 27980126

[ref16] van der Veen P. H. Muller M. Vincken K. L. Westerink J. Mali W. P. T. M. van der Graaf Y.. (2015). Hemoglobin, hematocrit, and changes in cerebral blood flow: the second manifestations of ARTerial disease-magnetic resonance study. Neurobiol. Aging 36, 1417–1423. doi: 10.1016/j.neurobiolaging.2014.12.019 25618615

[ref17] Vermeer S. E. Prins N. D. den Heijer T. Hofman A. Koudstaal P. J. Breteler M. M. B. (2003). Silent brain infarcts and the risk of dementia and cognitive decline. N. Engl. J. Med. 348, 1215–1222. doi: 10.1056/NEJMoa022066 12660385

[ref18] Walton B. L. Lehmann M. Skorczewski T. Holle L. A. Beckman J. D. Cribb J. A.. (2017). Elevated hematocrit enhances platelet accumulation following vascular injury. Blood 129, 2537–2546. doi: 10.1182/blood-2016-10-746479 28251913 PMC5418635

[ref19] Weiner D. E. Gaussoin S. A. Nord J. Auchus A. P. Chelune G. J. Chonchol M.. (2017). Cognitive function and kidney disease: baseline data from the systolic blood pressure intervention trial (SPRINT). Am. J. Kidney Dis. 70, 357–367. doi: 10.1053/j.ajkd.2017.04.021 28606731 PMC5572661

[ref20] Wolters F. J. Zonneveld H. I. Hofman A. van der Lugt A. Koudstaal P. J. Vernooij M. W.. (2017). Cerebral perfusion and the risk of dementia: a population-based study. Circulation 136, 719–728. doi: 10.1161/CIRCULATIONAHA.117.027448 28588075

[ref21] Wolters F. J. Zonneveld H. I. Licher S. Cremers L. G. M. on behalf of the Heart Brain Connection Collaborative Research Group Ikram M. K.. (2019). Hemoglobin and anemia in relation to dementia risk and accompanying changes on brain MRI. Neurology 93, e917–e926. doi: 10.1212/WNL.0000000000008003 31366722 PMC6745727

[ref22] Wood J. H. Kee D. B. (1985). Hemorheology of the cerebral circulation in stroke. Stroke 16, 765–772. doi: 10.1161/01.STR.16.5.765 3901420

[ref23] Wright C. B. DeRosa J. T. Moon M. P. Strobino K. DeCarli C. Cheung Y. K.. (2021). Race/ethnic disparities in mild cognitive impairment and dementia: the northern Manhattan study. J. Alzheimers Dis. 80, 1129–1138. doi: 10.3233/JAD-201370 33646162 PMC8150441

[ref24] Wright C. B. Festa J. R. Paik M. C. Schmiedigen A. Brown T. R. Yoshita M.. (2008). White matter hyperintensities and subclinical infarction: associations with psychomotor speed and cognitive flexibility. Stroke 39, 800–805. doi: 10.1161/STROKEAHA.107.484147 18258844 PMC2267752

