# Supplementary material for: Relationships of hematocrit concentration with dementia from a multiethnic population-based study
Source: Front Aging Neurosci. 2025 Feb 14;17:1543798. doi: 10.3389/fnagi.2025.1543798 (PMC11868278; doi:10.3389/fnagi.2025.1543798)
Supplement: Supplementary file 1 [file Table_1.docx]

Supplementary Table 1a: Full cox regression model output assessing hematocrit’s association with dementia risk

|  |  | P value | Hazard Ratio | 95% CI | |
| --- | --- | --- | --- | --- | --- |
| cox regression model |  |  |  |  |  |
| hematocrit as continuous |  | 0.037 | 0.952 | 0.909 | 0.997 |
| cox regression model |  |  |  |  |  |
| hct_mri | normal | ref |  |  |  |
|  | low | 0.046 | 1.807 | 1.012 | 3.227 |
|  | high | 0.156 | 0.701 | 0.429 | 1.145 |
| competing risk model |  |  |  |  |  |
| hct_mri | normal | ref |  |  |  |
|  | low | 0.208 | 1.405 | 0.798 | 2.473 |
|  | high | 0.238 | 0.712 | 0.419 | 1.209 |

Supplementary Table 1b: Full cox regression model output assessing hemoglobin’s association with dementia risk

|  |  | P value | Hazard Ratio | 95% CI | |
| --- | --- | --- | --- | --- | --- |
| cox regression model |  |  |  |  |  |
| hemoglobin | normal | ref |  |  |  |
|  | low | 0.032 | 1.539 | 1.038 | 2.282 |
|  | high | 0.624 | 0.703 | 0.172 | 2.87 |
| competing risk model |  |  |  |  |  |
| hemoglobin | normal | ref |  |  |  |
|  | low | 0.741 | 1.072 | 0.711 | 1.615 |
|  | high | 0.418 | 0.538 | 0.12 | 2.41 |

Supplementary Table 2: Interaction analyses

| Variable | Variable | P value |
| --- | --- | --- |
| sex | hct_mri_cat=1 | 0.8212 |
| sex | hct_mri_cat=2 | 0.1729 |
| ethnicity=1 | hct_mri_cat=1 | 0.7899 |
| ethnicity=1 | hct_mri_cat=2 | 0.9694 |
| ethnicity=2 | hct_mri_cat=1 | 0.4489 |
| ethnicity=2 | hct_mri_cat=2 | 0.9703 |
| apoe | hct_mri_cat=1 | 0.5962 |
| apoe | hct_mri_cat=2 | 0.4904 |
