# Supplementary figures and images for: Relationships of hematocrit concentration with dementia from a multiethnic population-based study
Source: Front Aging Neurosci. 2025 Feb 14;17:1543798. doi: 10.3389/fnagi.2025.1543798 (PMC11868278; doi:10.3389/fnagi.2025.1543798)

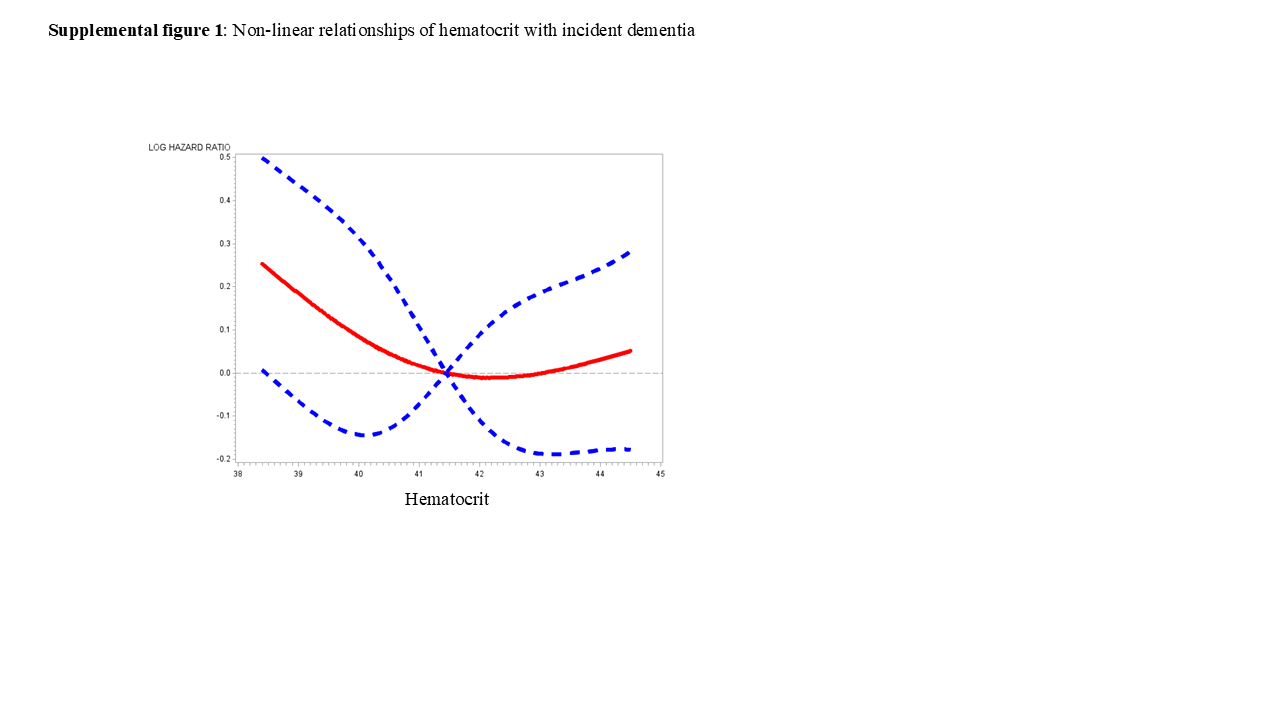

Supplement: Supplementary file 2 [file Image_1.tif]

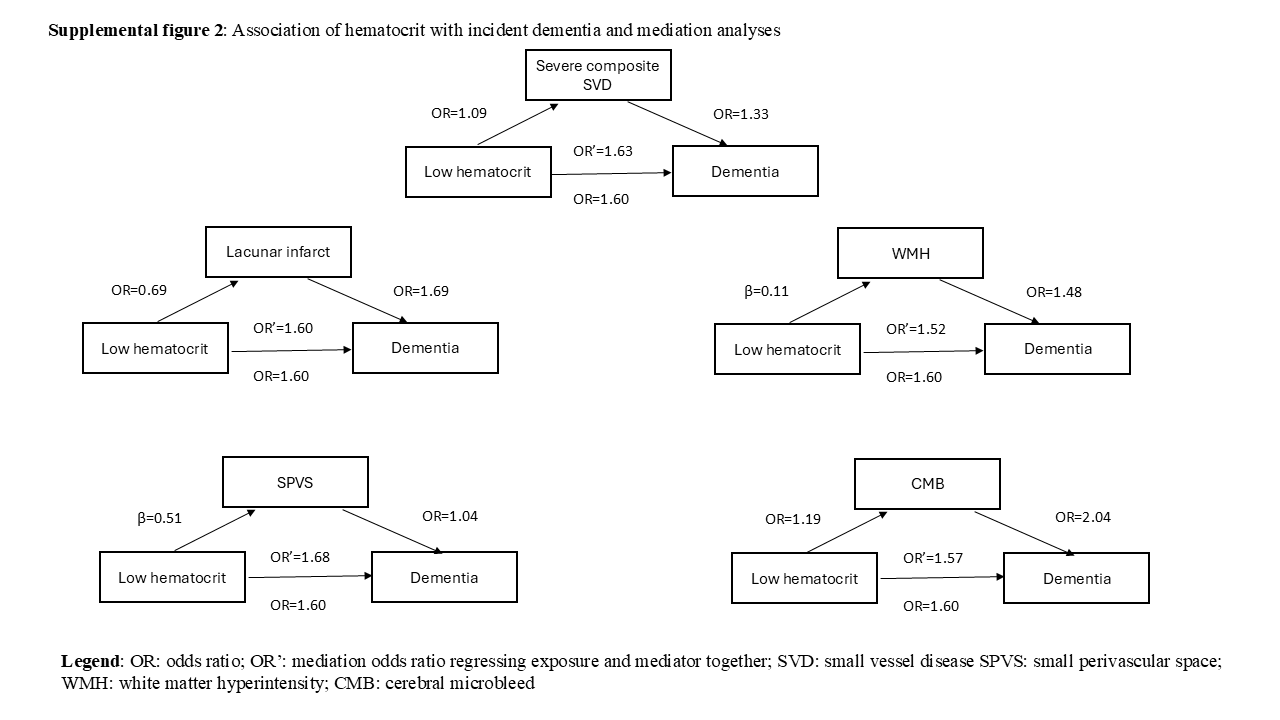

Supplement: Supplementary file 3 [file Image_2.tif]
